# Modeling causal conditional reasoning data using SDT: caveats and new insights

**DOI:** 10.3389/fpsyg.2014.00217

**Published:** 2014-03-12

**Authors:** Dries Trippas, Michael F. Verde, Simon J. Handley, Matthew E. Roser, Nicolas A. McNair, Jonathan St. B. T. Evans

**Affiliations:** Faculty of Health and Human Sciences, School of Psychology, Cognition Institute, Plymouth UniversityPlymouth, UK

**Keywords:** causal conditionals, reasoning, signal detection theory, belief bias, normative models

In deductive reasoning, people are asked to infer the truth of an argument's conclusion given a set of premises. Research into the processes underlying deduction has focused on examining how well people discriminate between logically valid and invalid arguments, and how irrelevant factors such as one's prior beliefs interfere with the ability to reason logically (Evans et al., 1983). This normative approach to validity has traditionally informed both practice and theory in the literature. However, its critics argue that “normativism” often leads investigators to biased or misleading interpretations of phenomena (Elqayam and Evans, 2011).

Formal modeling of deductive reasoning has often been successful by taking the traditional, normative approach. A case in point is the application of signal detection theory (SDT; Macmillan and Creelman, 2005) to the investigation of belief bias in syllogistic reasoning (Dube et al., 2010). In the SDT model, deductive judgments are based on strength of evidence; an argument is judged to be valid if its strength exceeds a criterion value. Because the choice of criterion is independent of the ability to discriminate between classes of arguments, the SDT model makes it possible to isolate response bias from accuracy. Dube et al. examined these two factors using ROC curves, which plot hits against false alarms at several levels of confidence. Hits and false alarms were defined in normative fashion as responding “valid” to logically valid and logically invalid conclusions, respectively.

Their analysis of ROCs led them to argue two significant points. First, contrary to prevailing theories of belief bias, conclusion believability can affect response bias without affecting the quality of reasoning. Second, the curvilinear shape of the ROCs is consistent with the distributional assumptions of SDT. The latter is a key test because finding linear rather than curvilinear ROCs would be problematic for the model. The curvilinear ROCs obtained in syllogistic (see also Dube et al., 2011; Trippas et al., 2013; but see Klauer and Kellen, 2010) and other forms of reasoning (Heit and Rotello, 2010, 2014) are similar to those widely observed in memory and perception (Pazzaglia et al., 2013). This consistency across domains strengthens the case for the usefulness of the SDT approach. It also leads to an expectation of similar findings in other areas of reasoning. Below, we describe findings from conditional reasoning that violate this expectation in a surprising yet enlightening way.

Causal conditionals are a form of deduction prevalent in everyday life. Consider the proposition: “If healthy foods are cheaper, then more people will eat healthy foods.” Four types of conditional inferences are possible: modus ponens (MP; “Healthy foods are cheaper, therefore more people will eat healthy foods”), modus tollens (MT; “Fewer people eat healthy foods, therefore healthy foods are not cheaper”), affirmation of the consequent (AC; “More people eat healthy foods, therefore healthy foods are cheaper”), and denial of the antecedent (DA; “Healthy foods are not cheaper, therefore less people eat healthy foods”).

From a normative point of view, MP and MT are valid and AC and DA are invalid inferences. Theories differ as to how people determine validity in these problems. According to mental model theory (Johnson-Laird and Byrne, 2002), people construct an initial mental model of the conditional (e.g., p q) which may then be fleshed out by considering additional models (not-p q; not-p not-q). According to the suppositional account of the conditional (Evans et al., 2003, 2002; Evans and Over, 2004, 2012), people evaluate the subjective probability of a conditional by hypothetically supposing p and then assessing the conditional probability of q given p, P(q|p). This relation between the natural language conditional and the conditional probability, P(if p then q) = P(q|p), can be used in a Bayesian/probabilistic model of conditional inference (Oaksford et al., 2000; Oaksford and Chater, 2009, 2013).

What these theories have in common is that there is no fundamental difference in how people process affirmation (MP + AC) and denial (MT + DA) inferences. This makes an SDT analysis straightforward and no different to that taken with the study of belief bias in syllogistic reasoning. For our case study, we analyzed aspects of a data set collected as part of a larger project under the direction of the fourth author of this paper^1^. This study examined the influence of belief in causal conditional problems (e.g., believable: “If oil prices continue to rise, then UK petrol prices will rise”; unbelievable: “If global temperatures rise, then less arctic ice will melt”). Hits were defined as “valid” responses to MP and MT and false alarms were defined as “valid” responses to AC and DA. This produced the ROCs seen in the top panel of Figure 1. The results are similar in some respects to those reported by Dube et al. (2010) for syllogisms: believability had no effect on accuracy (ROCs for believable and unbelievable items fall on the same curve) but seemed to affect response bias (confidence criteria for believable items are shifted to the right)^2^. However, there is a surprising difference: in contrast to the curvilinear ROCs observed with syllogisms, conditionals produced linear ROCs. A linear regression of the ROC (collapsing over believability) provided a good fit, *R*^2^ = 99.9%. Adding a quadratic component did not improve the fit, *p* = 0.78. Taken at face value, this result suggests that conditional reasoning requires a profoundly different model than the one that has seemed so successful when applied to other forms of reasoning, not to mention other cognitive tasks.

**Figure 1:**
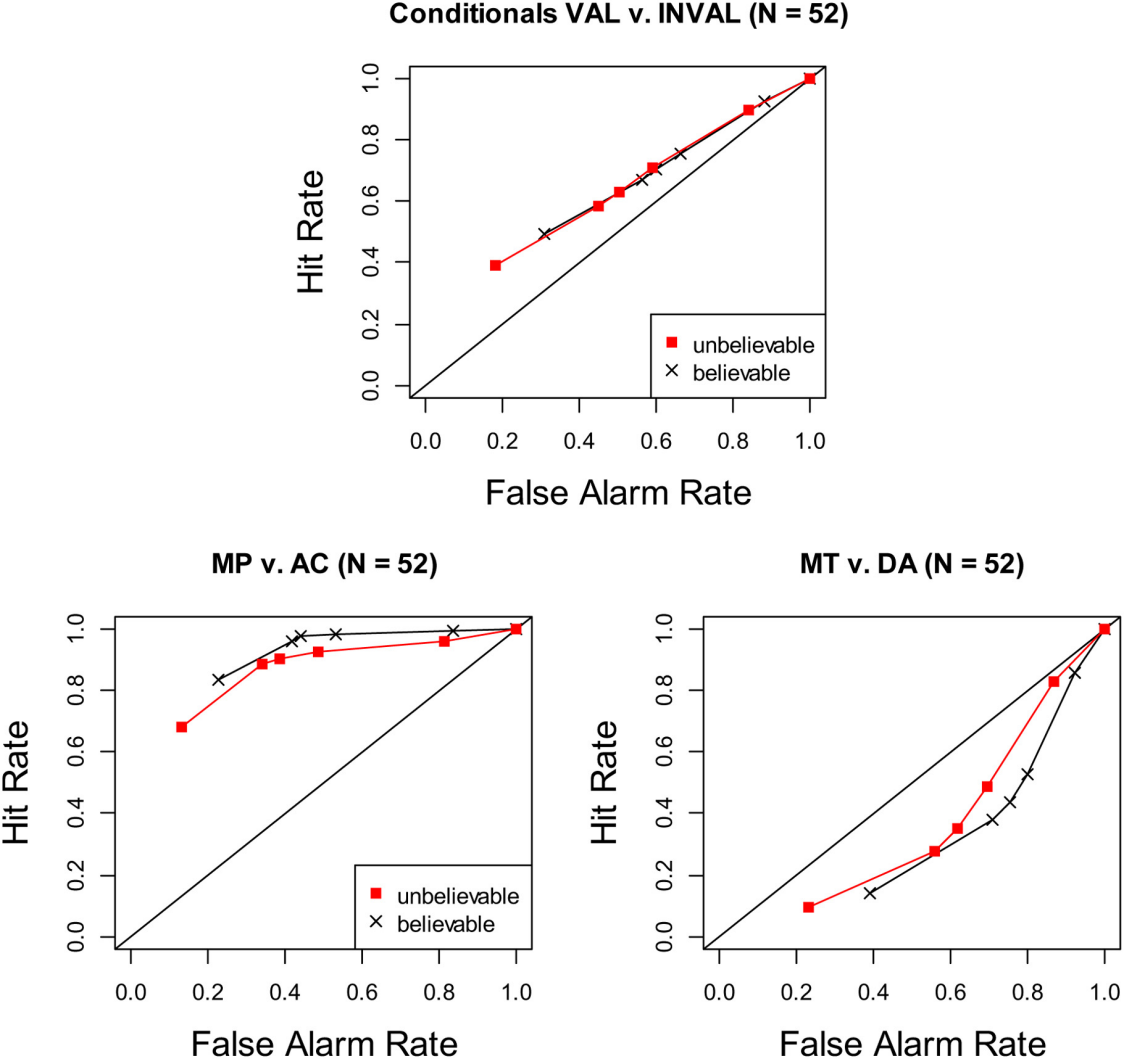
**ROC curves of causal conditionals. Top panel:** Valid (MP + MT) vs. invalid (AC + DA). **Bottom left:** affirmation conditionals (MP vs. AC). **Bottom right:** denial conditionals (MT vs. DA). Points on the ROC imply a more liberal response criterion (lower confidence responses) for identical levels of sensitivity. The points are plotted cumulatively such that the leftmost point = high confidence hits vs. false alarms, with the next point down being high + medium confidence hits vs. false alarms, and so forth.

A different picture emerges when we depart from the strictly normative approach and consider separately how people respond to affirmation and denial conditionals. In the bottom left panel of Figure 1, plotting MP (hits) against AC (false alarms) yields typically curvilinear ROCs. Linear regression (collapsing over believability) provided a fit, *R*^2^ = 96%, that was significantly improved by the addition of a quadratic component, *R*^2^ = 99.99%, *p* < 0.004. Accuracy is defined by the distance of the ROCs from the chance diagonal. Contrary to the poor accuracy on display in the aggregate results in the top panel, people are quite sensitive to argument structure when affirmation is involved. In the bottom right panel of Figure 1, plotting MT (hits) against DA (false alarms) again yields typically curvilinear ROCs. Linear regression (collapsing over believability) provided a fit, *R*^2^ = 98%, that was significantly improved by the additional of a quadratic component, *R*^2^ = 99.99%, *p* < 0.002. People were sensitive to argument structure, but the position of the ROCs below the diagonal indicates that their treatment of denial arguments departed from the normative; MT are treated as *less* valid than AC.

Applying the SDT model in a normative fashion, as would seem reasonable given extant theories of conditional reasoning, produced results that contrast sharply with previous findings. The clearly linear ROC in the top panel of Figure 1 is not only unlike the curvilinear ROCs observed with syllogisms but if taken at face value is problematic for the SDT model. It could be that there is something fundamentally different in the way that people reason about causal conditionals as compared to other types of problems. It seems to us more likely that the difference lies with affirmation and denial inferences; the latter do not seem to be treated in the normatively prescribed fashion. Once this is assumed, the ROC results become more sensible and fall in line with previous results (in a reanalysis of published and unpublished data sets, Heit and Rotello, 2014, have also reported curvilinear ROCs from MP plotted in the manner of Figure 1, lower left). This interpretation converges with Singmann and Klauer's (2011) finding, based on state-trace analysis, that affirmation and denial problems may depend on different processes.

Why use ROC analysis rather than simply examine the raw validity judgments? Interpreting the latter often relies on assumptions that may not be justified (Klauer et al., 2000; Dube et al., 2010). The main advantage of a formal model like SDT lies in its specification of assumptions. However, models can also produce insights that are not obvious at first glance. A qualitative difference between affirmation and denial inferences is not necessarily predicted by extant theories. Moreover, various manipulations seem to exert a similar effect on both types of inferences (e.g., Cummins, 1995). Finally, it is interesting to note that the production of linear ROCs when performance is driven by multiple underlying processes has been predicted in theory (DeCarlo, 2002). These results may offer a case study of how this can occur in practice.

## Footnotes

^1^This research was supported by the award of an ESRC project grant RES-062-23-3285.

^2^Note that the current data pattern does not necessitate a criterion shift, but that it is also consistent with a symmetrical distribution shift. For more discussion on this issue, see, e.g., Verde et al. (2010).
